# BNT162b2 COVID-19 Vaccine Hesitancy among Parents of 4023 Young Adolescents (12–15 Years) in Qatar

**DOI:** 10.3390/vaccines9090981

**Published:** 2021-09-02

**Authors:** Sarah Musa, Ismail Dergaa, Mariam Ali Abdulmalik, Achraf Ammar, Karim Chamari, Helmi Ben Saad

**Affiliations:** 1 Primary Health Care Corporation (PHCC), Doha P.O. Box 26555, Qatar; idergaa@phcc.gov.qa (I.D.); mabdulmalik@phcc.gov.qa (M.A.A.); 2 Institute of Sport Science, Otto-von-Guericke University, 39104 Magdeburg, Germany; achraf1.ammar@ovgu.de; 3 Interdisciplinary Laboratory in Neurosciences, Physiology and Psychology: Physical Activity, Health and Learning, UFR STAPS, UPL, Paris Nanterre University, 92000 Nanterre, France; 4 Aspetar, Orthopaedic and Sports Medicine Hospital, FIFA Medical Centre of Excellence, Doha P.O. Box 29222, Qatar; karim.chamari@aspetar.com; 5 Heart Failure Research Laboratory (LR12SP09), Farhat HACHED Hospital, University of Sousse, Sousse 4054, Tunisia; helmi.bensaad@rns.tn

**Keywords:** asymptomatic, herd immunity, mRNA, public health, vaccine acceptance

## Abstract

Parental vaccine hesitancy (VH) remains a barrier to full population inoculation, hence herd immunity against the SARS-CoV-2 virus. We aimed to determine parental VH rate, subgroups and influencing factors related to the BNT162b2 COVID-19 vaccine among their young adolescents (12–15 years old) in Qatar. A retrospective, cross-sectional study was conducted from 17 May to 3 June using vaccination booking records of 4023 young adolescents. Sociodemographic characteristics (i.e., age, sex, and nationality), health status and BNT162b2 COVID-19 vaccination booking status were analysed. Among respondents, the VH rate was 17.9%. Parents of 12-years adolescents were more hesitant (21.6%) as compared to the 13- (16.0%) and 15- (15.2%) years groups (*p* < 0.05). Parents of adolescents belonging to Gulf Countries (97% Qatari) were more hesitant (35.2%) as compared to the four remaining groups of nationalities (Asiatic; excluding Gulf Countries), North-African, African (excluding North-African), and European/American/Oceanian, 13.3–20.4%, (*p* < 0.001). Parental VH rates were higher when adolescents suffered from chronic disease as compared to those without the chronic disease (21.3% vs. 17.4%, *p* < 0.05) or who previously were COVID-19 infected as compared to non-previously COVID-19 infected (24.1 vs. 17.5%, *p* < 0.01). Results of logistic regression revealed that age groups, nationalities, and recovery from COVID-19 were the main predictors of VH level. Precisely, parents of 12 years old adolescents were 38% more likely to be hesitant as compared to the parents of the 15 years old adolescents (OR = 1.38; 95%CI: 1.12–1.70). Compared with the Gulf countries, parents of adolescents belonging to the other nationality categories; namely North-African, African, Asiatic and European/American/Oceanian were 48% (95%CI: 0.36–0.65), 41% (95% CI: 0.27–0.62), 38% (95%CI: 0.29–0.50) and 34% (95% CI 0.21–0.56) less likely to be hesitant, respectively. Furthermore, parents of young adolescents being previously COVID-19 infected were 37% more likely to be hesitant as compared to those with no previous COVID-19 infection (OR = 1.37; 95%CI: 1.02–1.84). Effective communication strategies specifically targeting Gulf Country populations, parents of younger children aged 12 years and of those with chronic disease or have been previously infected with COVID-19 are crucial to build community trust and vaccine confidence, thereby increasing COVID-19 vaccine uptake.

## 1. Introduction

Over 216 million people confirmed cases of severe acute respiratory syndrome coronavirus (SARS-CoV-2) and more than 4.5 million associated fatalities; have been reported globally since the pandemic began, up to 28 August 2021 [1]. In efforts to contain the spread of COVID-19, strategic preparedness and response plan guided by the World Health Organization (WHO) in collaboration with international governments and authorities was initiated rapidly [2,3]. Widespread population level physical distancing measures and movement restrictions were enforced to mitigate the transmission of the virus [4,5]. In Qatar, a mass immunization campaign was launched on 23 December 2020, that primarily encompassed the ‘at risk’ population including people over 70-year, people with chronic conditions and key healthcare workers, and later progressively included younger individuals.

Epidemiological data show that COVID-19 in the age group 1–18 years accounts for marginal proportion, tend to be asymptomatic, and overall, present significantly more favourable outcomes than in adults [6]. Despite a better prognosis and low mortality in children/adolescents, the disease can progress to multisystem inflammatory syndrome, a life-threatening complication of COVID-19 infection [7]. The cumulative COVID-19–associated children/adolescent hospitalization rate in 99 countries across 14 states in the US was 12.5 times lower than that in adults aged ≥18 years [8]. Besides that, figures of weekly COVID-19–associated children/adolescent hospitalization rates were comparable to rates among preschool children aged 0–4 years, but higher than rates among children aged 5–11 years [8]. Nevertheless, with increased incidence of asymptomatic carriage and milder symptoms, children/adolescents are likely to become a silent reservoir of infection and remain a threat, as a significant source of enduring transmission [9].

Vaccination is frequently cited as one of the most effective ways to prevent and control infectious diseases [10]. A growing body of evidence indicates that people fully vaccinated with an mRNA vaccine (BNT162b2 or mRNA-1273) are less likely to have symptomatic infection or to transmit severe acute respiratory syndrome coronavirus 2 (SARS-CoV-2) to others compared to unvaccinated individuals [11]. Furthermore, these vaccines have shown effectiveness in reducing the rate of hospitalizations, severe outcomes, and related mortality [11]. Globally, mass vaccination campaigns against SARS-CoV-2 infection were primarily made available for adults, far ahead. By 10 May 2021, the U.S.A Food and Drug Administration (FDA) has expanded the authorization of BNT162b2 vaccines to embrace young adolescents 12 through 15 years of age [12]. Thereafter, in a clinical trial founded by Pfizer, it has been reported that the BNT162b2 vaccine in 12-to-15-year-old recipients had a favourable safety profile, produced a greater immune response than in young adults, and was highly effective against COVID-19 [13]. In Qatar, the Ministry of Public Health (MOPH) has, therefore; launched a vaccination campaign amongst this age group on the 12 of May 2021, i.e., two days following the U.S.A. FDA approval and authorization for this age group.

Vaccinating children/adolescents is seen as crucial to ending the pandemic by helping the world obtaining remarkable population coverage to acquire herd immunity. However, vaccine hesitancy (VH) which refers to the delay in acceptance or refusal of vaccines despite availability of vaccine services [14] remains a barrier to full population inoculation. Thus, the development of effective vaccine communication strategies is imperative to promote public confidence, leading to increased vaccine acceptance. A survey of over 5000 families from Bologna Italy, has reported a high rate of parental VH against SARS-CoV-2 in their children and adolescents aged <18 years old (40%) most notably detected among female parents of younger children age (6–10 years), parents aged ≤29 years old, with low educational level, mainly relying on information found in the web/social media and disliking mandatory vaccination policies [15]. Although this study highlighted specific groups that could benefit from targeted public health awareness campaigns, these findings should be interpreted with caution as to their representability of other regions of the globe. Indeed, this large survey was carried out at the very beginning of the Italian anti-COVID-19 vaccination campaign (between December 2020 and January 2021) when the public debate on this topic was not yet politicized or polarized and most importantly, even children/adolescents’ vaccination was not yet considered, let along authorized. Therefore, updated specific data following the official authorization of children/adolescents’ vaccination is warranted.

In this context, a recent review highlighted that the overall COVID-19 VH for adults differs between nations [16]. Thus, it seems also interesting to understand the effect of the nation of origin on parental hesitancy toward children/adolescents’ vaccination through multicentre studies or studies in countries with a multinational population (such as Qatar, an Arabic Gulf Country in the Middle East, belonging to the Gulf Cooperation Council). Therefore, the aims of this study were (i) to determine the parental hesitancy rate of the BNT162b2 COVID-19 vaccine for their young adolescents (12–15 years old), and (ii) to investigate factors that might influence the VH rate in Qatar.

## 2. Methods

### 2.1. Study Design and Participants

This was a retrospective cross-sectional study of acceptance and hesitancy rates of the COVID-19 vaccine among parents of adolescents. In the overall cohort, we included young adolescents 12 to 15 years of age from Rawdat Al Khalil Health Centre (RAK-HC). The study used data collected from 17 May to 3 June 2021. RAK-HC is a leading governmental hospital in Qatar, dedicated as a COVID-19 institution since the beginning of the pandemic and has been the primary receiver of patients suspected of COVID-19 in Qatar.

**Figure 1:**
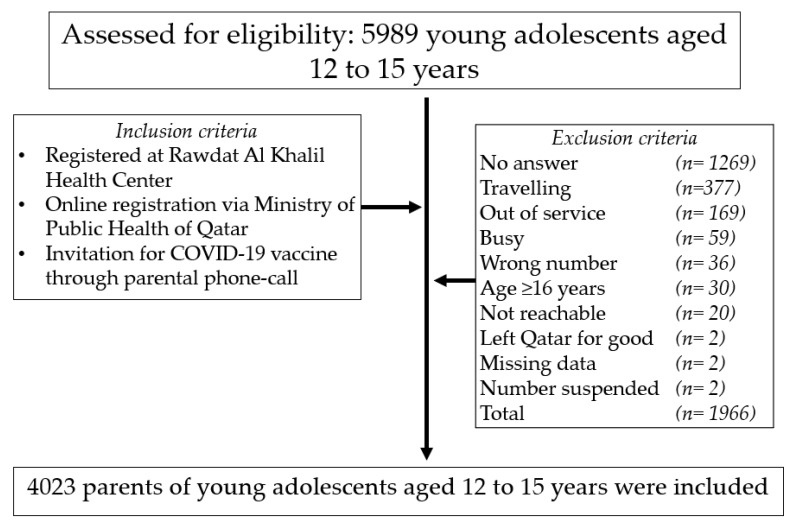
Study flowchart.

A list of eligible participants was obtained from two domains: (i) a database of adolescents aged 12–15-year-old registered at RAK-HC central region, or (ii) online registration via the MOPH website, in which parents were able to book vaccine appointments remotely for their adolescents. The exclusion criteria are highlighted in Figure 1. The study was exempted from Institutional Review Board approval and informed consent was waived off due to the retrospective nature of the study. However, the study was granted data release approval from the department of clinical research-Primary Health Care Corporation (PHCC) under the reference (PHCC/DCR/05/036). Anonymized data were obtained from PHCC medical record-health information system.

### 2.2. Vaccination Campaign in Qatar

In Qatar, the National COVID-19 vaccination campaign was first launched on 23 December 2020 after FDA authorization of Pfizer-BioNTech COVID-19 vaccine on 11 December 2020. Pfizer-BioNTech was made available in Qatar from December 2021 and was eligible for people aged 16 years and older, while the Moderna vaccine for 18 years and over was rolled out from the 2nd week of February 2021.

The vaccine was offered by invitation only, and according to the following four-phased vaccination plan:

- Phase 1 (from 23 December 2020 to 31 March 2021) encompassed people who are aged 70 years and above, long-term care and home care patients, people with severe or multiple conditions including age group from 16 years and older, key personnel and first responders including health care providers, ambulance teams, Ministry of Interior, Ministry of Defence, oil and gas sector, Hamad International Airport and Qatar Airways, and 50% of teachers.
- Phase 2 (from 1 April to 30 June 2021) encompassed people aged 40 years and above, those with moderate health conditions, workers in industries including food, housekeeping, hairdressing and transportation, all remaining teachers, all health care personnel not included in the first stage, people in group accommodation facilities for persons with physical disabilities or psychological problems or in the recovery phase and persons in detention facilities. Throughout phase 2, young adolescents aged 12–15 years old were added on 12 May 2021, two days following the U.S.A. FDA approval and authorization of Pfizer-BioNTech COVID-19. Adolescents aged 16–17 who were not vaccinated in phases 1 and 2 were included as well.
- Phase 3 (from 1 June to 31 July 2021) included workers in industries essential to the functioning of society who are at increased risk of disease and eligible people who were not included in the first and second phases.
- Phase 4 (from 1 August to 31 October 2021) intends to include all the residents of Qatar aged 12 years and more, who did not receive the vaccine in the previous stages.

As of 18 May 2021, more than 2.1 million vaccine doses have been administered since the start of the vaccination campaign with 53.8% of the eligible population having received at least one dose of the vaccine. For the population over 60, 89.2% have been vaccinated with at least one dose, and 83.7% have received both doses [17].

### 2.3. Sample Size

To avoid selection bias and enhance the generalizability of our results, we aimed at collecting a large sample in a single vaccination centre where all young adolescents (12–15 years) who were registered at RAK-HC and were invited for COVID-19 vaccination booking between the period of 17 May to 3 June 2021, were eligible for enrolment (*n* = 5989). After applying the exclusion criteria shown in Figure 1, 4023 participants were included in the current study.

### 2.4. Vaccine Acceptance and Hesitancy

Vaccine acceptance refers to a parental agreement to obtain a confirmed COVID-19 vaccine booking appointment for their adolescents at the time of call invitation. Vaccine acceptance is not synonymous with actual vaccine uptake, which is not a dependent variable utilized in this study.

VH refers to delay in acceptance, or hesitancy of vaccines despite availability of vaccine services [18]. VH is complex and context-specific, varying across time, place, and vaccine’s types/brands. It is influenced by factors such as complacency, convenience, and confidence [18]. The VH determinants matrix displays the factors influencing the behavioural decision to accept, delay or reject some or all vaccines under three categories: contextual, individual and group, and vaccine/vaccination-specific influences [18].

### 2.5. Data Collection

Parents of young adolescents were categorized into two main groups according to vaccine booking status: accepted or hesitant. For parents with multiple eligible adolescents, each response was handled and counted as a distinct participant irrespective of the sibling’s number.

Data related to adolescents’ sociodemographic characteristics (i.e., age, sex, nationality), health status (i.e., history of any previous COVID-19 infection during the last 90 days previous to the call/booking, and if at high-risk category i.e., having one or more comorbidities) in addition to vaccine status (acceptance or hesitancy) and VH reasons (i.e., “wait and see”, “definitely refused”, “COVID-19 positive” or “currently sick” at the time of call/booking) conveyed by parents were extracted from the database maintained in an Excel sheet. Nationalities were arbitrarily classified into five groups: (i) Asiatic (excluding the second group), (ii) From the Gulf Countries (Bahraini, Emirati, Kuwaiti, Omani, Qatari, and Saudi Arabian), (iii) North-African (Algerian, Egyptian, Libyan, Mauritanian, Moroccan, and Tunisian), and (iv) African (excluding the third group), and (v) European, American & Oceanian. Young adolescents of hesitant parents were followed up for at least one month after the initial invitation to access if there would be any change in their vaccination status.

### 2.6. Statistical Analysis

Age was presented as mean ± standard deviation and categorical data were expressed as number and %. The chi-2 test was used to compare categorical data between two groups (sex, presence of chronic disease, being previously COVID-19 infected), and more than two groups (four age groups and five nationality groups). Factors affecting parental VH (i.e., sociodemographic characteristics and health status) were also analysed by binary logistic regression, with odds ratios (ORs), 95% confidence intervals, and *p*-values ≤ 0.05 calculated for each independent variable. The dependant variable was “vaccine booking status” and “vaccine acceptance” was the reference category. During the determination of the reasons for parental VH, and in front of a low number of adolescents qualified as “currently sick” (*n* = 5), the reasons “currently sick” and “COVID-19^+ve^” were merged into a single group. All mathematical computations and statistical procedures were performed using statistical software (Statistica, version 12). The significance level was set at 0.05.

## 3. Results

Out of the 5989 parents of young adolescents, 4023 were included in this study. The data of the remaining 1966 parents of young adolescents were excluded from the analysis for several reasons described in Figure 1.

### 3.1. Parents and Young Adolescents’ Characteristics

The adolescents mean ± standard deviation age was 13.4 ± 1.1 years. Table 1 exposes the characteristics of the parents and young adolescents. The adolescents’ age groups 12 and 13 years dominated the age groups from a number perspective. Similar percentages of boys and girls were included in the total sample (*p* = 0.2028). Asiatic and North-African nationalities represented together 84.42% of the total population, and the Qatari nationality represented 7.58% of the total population and 97% of the total Gulf Countries. Adolescents with at least one chronic disease and/or who have been previously COVID-19 infected represented 12.50% and 6.29% of the population, respectively.

Seventy-one nationalities were included (Table S1. The five most presented nationalities were: Indian (28.57%), Egyptian (17.35%), Filipino (9.72%), Qatari and Jordanian (7.58% each). For nationalities including 10 young adolescents or more, the VH rates ranged from 7.58% (Pakistani) to 40.91% (Algerian) (Table S1).

### 3.2. Influencing Factors of Vaccine Acceptance and Hesitancy Rates

The vaccine acceptance (82.10%) and hesitancy (17.90%) rates were statistically different (*p* < 0.001). As shown in Table 1, the vaccine acceptance rates were significantly lower in:

(i). The 12 years group compared to the 13- and 15-years groups,
(ii). North-African nationalities compared to Asiatic nationalities,
(iii). Gulf Countries nationalities compared to the four remaining groups of nationalities,
(iv). Young adolescents with the chronic disease compared to those free from chronic disease, and in
(v). Young adolescents having been previously COVID-19 infected as compared to their COVID-19 negative-history counterparts.

The results of multivariate analysis (binary logistic regression, Table 1) indicated that age groups, nationalities, and recovery from COVID-19 significantly predicted the level of VH. Parents of 12 years old adolescents were 38% more likely to be hesitant as compared to the parents of the 15 years old adolescents. Compared with the Gulf Countries, parents of adolescents belonging to the “Asiatic”, “North-African”, “African” and “European/American/Oceanian” nationality categories were 34 to 48% less likely to be hesitant. Furthermore, parents of young adolescents being previously COVID-19 infected were 37% more likely to be hesitant compared to those with no previous COVID-19 infection.

### 3.3. Reasons for Parental VH

As shown in Table 2, the main reason for parental VH was “wait and see” in 509 cases (70.69%). Adolescents’ age groups, sex and nationalities did not influence the VH reasons. However, the presence of chronic disease has significantly influenced VH reasons with most reporting ‘wait and see’, while no statistically significant difference was found for ‘definitely refusal’. In addition, a statistically significant difference was found in reporting “definitely refusal” or “Currently sick or COVID-19^+ve^” as reasons of VH between participants who have been recovered from COVID-19 and who have not had previous COVID-19 history.

**Figure 2:**
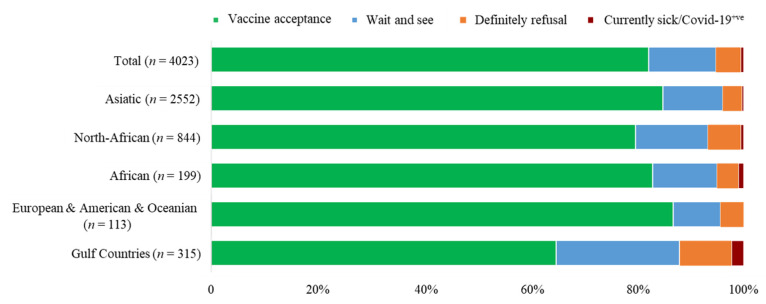
Percentage of BNT162b2 COVID-19 vaccination acceptance and hesitancy subcategories among parents of young adolescents by groups of nationalities.

Figure 2 demonstrates the percentages of BNT162b2 COVID-19 vaccination acceptance and hesitancy subcategories among parents of young adolescents by groups of nationalities. Among all nationalities, respondents from North-Africa and Gulf Countries have declared the highest rate of “Definitely refuse” (27.8% and 16.58%, *p* < 0.001 respectively).

### 3.4. Vaccine Acceptance among Previously Vaccine Hesitant Parents

Twenty-three percent of previously VH parents have changed their vaccination status to accept up to the end of June 2021. The majority of these parents’ young adolescents have already received at least one dose of COVID-19 (66%) while (34%) had an appointment booked at the time of data processing (10 July 2021).

## 4. Discussion

To the best of the authors’ knowledge, this is the first study around the world to investigate the rate and predictors of parental hesitancy related to the BNT162b2 COVID-19 vaccine for their 12 to 15 years old adolescents, following its emergency use authorization among this age group (10 May 2021). Results of this study would provide insight into the potential determinants of vaccine hesitancy among parents of young adolescents providing useful preliminary information to plan future children/adolescents COVID-19 vaccination campaigns. The main result of this study was the associations between the young adolescents’ characteristics (i.e., age, nationalities and health status) and the parental VH, with a higher rate of VH in parents of adolescents aged 12 years old, living with chronic disease or with COVID-19 history and/or belonging to Gulf Countries (e.g., Qatar).

Willingness to receive the COVID-19 vaccine whenever it gets authorisation for emergency use in children/adolescents was looked at by several studies in the initial phase of vaccine manufacturing [8,15,19]. However, several ongoing clinical trials were examining various types of vaccines, and together with the lack of public data especially amongst the 12–15 years old age group, greater hesitancy and doubts have been encountered. For instance, given the sizable gap between intentions and behaviours, the actual vaccine uptake by confirmed booking appointment would not be comparable to vaccine willingness, particularly prior to authorization.

### 4.1. VH amongst Parents of Young Adolescents According to Age Groups and Sex

Our findings revealed a low VH rate of 17.9% amongst the whole population. The main reason for VH was “wait and see” (70.69%). There was a significant effect of adolescents’ age on parental VH. Parents of 12-years adolescents were more hesitant as compared to the 13-years and 15-years groups to vaccinate their children. While we revealed a significant difference between vaccine acceptance and hesitancy rates with regard to adolescents’ age, there was no difference for sex. In contrast, Simonson et al. [19] in their large-scale study (*n* = 19,789) across 50 states in the USA, reported that children’s age did not play a role in parental COVID-19 VH. Parents of children (up to 12 years old) and adolescents (13–17 years old) showed about the same level of hesitancy (around 20%) to vaccinate their children [19]. High rates of parental VH encompassing, both children and adolescents could be driven by a combination of fear and complacency over the perceived risks, or low perception of infection susceptibility possibly due to parents being more protective and exert further control in terms of social gathering over younger adolescents.

### 4.2. VH amongst Parents of Young Adolescents According to Nationalities

Parents belonging to Gulf Countries, mostly native Qatari (97%), were more hesitant in getting their young adolescents vaccinated as compared to the four remaining groups of nationalities. Limited studies have assessed parental vaccine acceptance toward COVID-19 vaccination in the paediatric population, showing extremely heterogeneous results [i.e., 72.6% (China [20]), 75.8% (Australia [21]), 48.2% (the UK, where 40.9% of parents responded as “unsure but leaning towards yes” [22])]. The wide heterogeneity in the VH rate reported by the various studies [20,21,22] is not surprising. The VH phenomenon is known to be of great complexity, as it results from the confluence of several demographic, socio-economic, cultural, and/or geographic factors [18]. Previous studies have shown that media-driven conspiracy about COVID-19 has emerged as a common phenomenon globally, with variability in rates amongst diverse nationalities. For instance, COVID-19 VH rate for adults was 5.7% in Malaysia [23], 6.7% in China [24], 18.4% in South Africa [25], 21.0% in UK [16], and 25% in USA [26].

Importantly, the present study showed that compared with parents of adolescents belonging to the Gulf Countries, immigrant parents were 34% to 48% less likely to be hesitant. These findings are consistent with a recent study by Alabdullah et al. [27] examining COVID-19 VH and attitudes for adults in Qatar and illustrating that native Qataris were more likely to be vaccine hesitators (42.57%) compared to the immigrant population (16.71%). In our study, the higher acceptance rate among non-Qataris could be interrelated to imposed travel policies and exemption of quarantine that rendered travel easier to home countries, especially during the summer holidays. COVID-19 VH was higher in Arab countries in previous studies; Saudi Arabia (35.28%) [28], Jordan (62.6%) [29], and in a small multinational survey that included several countries, mainly Jordan and Kuwait (70.6%) [30]. Similarly, a large-scale study (*n* = 36,220) from 23 Arab countries and 122 other countries was conducted from 14 to 29 January 2021. It revealed substantial VH rates among Arabs and outside Arab regions (83% and 81%, respectively) [30]. The most cited reasons for VH were worries about side effects and distrust in healthcare policies, vaccine expedited production, lack of trust in both the published studies and vaccine producing companies [30]. Additionally, Qunaibi et al. [31] noted that female participants, those aged 30–59-year-old, those with no chronic diseases, those with a lower level of academic education, and those who lack knowledge about the type of vaccine authorized in their countries were more hesitant to receive COVID-19 vaccination. On the other hand, it has been reported that participants who regularly received the influenza vaccine, health care workers, and those Qatar’ residents coming from countries with higher rates of COVID-19 infections showed more vaccination willingness [32].

Early-stage vaccination resistance could be attributed to the fear of side effects or mistrust/misinformation related to the vaccine, a possible explanation that might lead the majority of parents from Gulf countries to be more resistant in vaccinating their young adolescents, especially that holiday travels were not seen as necessary in comparison to other nationalities for their intentions of returning to their home countries (particularly after summer 2020 during the first phase of the pandemic, when many expatriates chose not to fly out due to the associated complexity and risks). Parents probably need more reassurance over time especially as the authorisation of the vaccine by the FDA was released just two days preceding the commencement of the ‘’12–15-year of age population’’ vaccination campaign in Qatar. Another contributing factor could also be related to rumours, misinformation received from media or acquaintances. Moreover, Gulf countries are small countries with shared beliefs and culture between citizens perhaps easily influenced by other residents and local social media. Nevertheless, local public health strategies should aim to provide further evidence-based updates on vaccine effectiveness and safety among children/adolescents to promote vaccine confidence. Health care authorities shall consider setting the channels of communication with the communities via physicians as per their credibility amongst the people [33].

The higher level of acceptance among non-GC residents may be linked to the number of COVID-19 cases and deaths in their respective home countries which might contribute to a higher perception of risk and severity, influencing the need for self and family protection. However, further studies are indeed required to confirm this hypothesis. Another explanation could be the availability of free and highly effective COVID-19 vaccines in Qatar, which would be a handy choice for people, particularly those from low-income countries where vaccine availability is limited. Countries with higher socioeconomic status have been connected to higher educational levels, a correlating factor that might increase their exposure to social media, hence enhancing the further spread of misinformation and fear.

### 4.3. VH amongst Parents of Young Adolescents Living with a Chronic Disease

Our findings revealed that parents of young adolescents with a chronic disease, despite a low VH rate (21.27%) showed remarkable lower confidence in the BNT162b2 COVID-19 vaccine as compared to parents of young adolescents without any chronic disease. Contrary to our findings, a survey on COVID-19 acceptance and hesitancy rates among ≥18 years old in Italy [34] reported that respondents with no history of comorbidities were two times more likely to be vaccine-hesitant (OR = 1.95: 1.36–2.80). Such results provided empirical insight about community awareness/culture, risk, and benefit perception among this study cohort. Although the mortality rate associated with COVID-19 remains low among children, the risk of severe COVID-19 infection is most encountered among those vulnerable children with underlying comorbid conditions such as obesity, asthma, sickle cell disease, and immunosuppression [35]. Therefore, the potential link between COVID-19 severity and patient vulnerability level (or health status) may raise parental awareness regarding the need of immunising adolescents with chronic diseases, hence promoting vaccine confidence. Nevertheless, it has been reported that greater resistance to vaccination seen in participants with existing chronic health problems may be explained by the presence of individuals for whom vaccines are medically contraindicated and/or by a fear of iatrogenic effects of a vaccine among these individuals [36]. A low level of knowledge related to vaccine eligibility in adolescents living with diverse chronic medical conditions as well as lack of trust in vaccine safety within compromised immune system individuals together with likely concerns related to drug interaction may justify the higher VH among this category in our study. Integrated care plan provided by educational intervention tackling the most dominant source of patient’s information (mainly physicians) would probably improve public and parental awareness about the risks and benefits ratio and hopefully translate fear into opportunities. If infected with COVID-19, ‘high risk’ teenagers with underlying chronic illnesses that result in immunosuppression are more likely to develop severe illness than adults. Those adolescents are at a higher risk of being admitted to the hospital, being admitted to critical care, needing a ventilator, or possibly dying. COVID-19 may raise the risk of severe illness in children and adolescents with medical complexity, genetic, neurologic, metabolic, and/or congenital heart disease, according to previous research [37]. Furthermore, after contracting COVID-19, young adolescents can acquire long-term symptoms known as long COVID-19 or multisystem inflammatory syndrome-children [38]. As a result, it was advised that for children aged 12–17 years who are in medical risk groups, the benefits of immunization with the BNT162b2 vaccine outweigh the risks of probable adverse effects [38].

### 4.4. VH amongst Parents of Young Adolescents Previously Infected by COVID-19

Parents of young adolescents having recovered from COVID-19, despite a low VH rate (24.11%) were more hesitant as compared to COVID-19 negative-history counterparts. In line with our results, a previous study by Gerussi et al. [39] reported that one out of three patients having recovered from COVID-19 infection were undecided regarding the COVID-19 vaccine. Among patients who justified their choice, 20.8% considered themselves in no need for vaccination due to their young age, health status or believed that immunization post-COVID-19 infection was enough to protect them; others manifested doubts on vaccine efficacy (18.8%). As explained by the authors, such findings could be related to the patients’ negative recent disease experience, lack of public information about the COVID-19 vaccine, in addition to their belief in immunity post-infection, as pointed out in multiple surveys on VH among people after H1N1 [40,41]. At the beginning of the pandemic, immunity to COVID-19 was poorly known as well as the data regarding reinfection. Being a potentially formerly infected parent as well as the influence of low perceived susceptibility will indeed be reflected on decision and acceptance of vaccination in their children. Furthermore, throughout the first wave of the pandemic, severity and mortality of the disease was linked to older age and the coexistence of chronic conditions, potentially leading to overconfidence concerning younger age patients [42]. The limited availability of research in comparing vaccine- and natural- induced immunity in terms of COVID-19 infectivity/transmission or antibody response (durability and effectiveness) could also be a contributing factor to VH among previously COVID-19 positive individuals [40].

## 5. Hesitancy Reasons of the BNT162b2 COVID-19 Vaccine amongst Parents of Young Adolescents

Our results revealed that age groups, sex and nationalities did not influence the VH reasons (Table 2). Yimazy et al. [43] identified that amongst the reasons that influence parental willingness to vaccinate their children were the need for COVID-19 control (75.5%), the benefits of the COVID-19 vaccine outweighing its potential harm (69.9%) and to protect not only their own families but also others (66.2%). Some surveys have identified that views on COVID-19 vaccine acceptance are influenced by vaccine efficacy and perceptions of disease risk. In a cross-sectional survey conducted in Indonesia, Harapan et al. [44] highlighted that 93.3% of participants would receive a COVID-19 vaccine that was 95% effective but only 67.0% of participants would accept a 50% effective vaccine. The authors also noted that the vaccine acceptance rate was higher amongst participants that considered themselves at greater risk of COVID-19 infection.

Parents are usually the decision-makers, so it is important to understand initially the parental acceptance rate of COVID-19 vaccination to guide national efforts toward awareness and promotion strategies as well as the preparedness of health system capacity. A possible explanation could be addressed by the health belief model [45] that proposes multiple factors influencing health behaviours and vaccination intentions, namely perceived susceptibility of severity or benefits or barriers, cues to action and individual characteristics or self-efficacy. According to Yilmaz et al. [45], parents who received positive information related to the COVID-19 vaccine in social media were more likely to vaccinate their children (39%). The main reasons for reluctance to allow their children to receive the COVID-19 vaccine included lack of sufficient scientific studies (84.8%), concern about safety and side effects (76.9%), and potential inefficacy of the vaccine due to mutations (36.7%). Correspondingly, Marquez et al. [44] found that 39.2% of caregivers refused to vaccinate their children with COVID-19 vaccination, whereas 27.8% agreed that if their physician recommended the COVID-19 vaccine they would allow their children to receive it. In the latter study, up to 52.2% of participants claimed that a health care professional could influence their decision. This places physicians and health care professionals at an important and pivoting role in public awareness of vaccination role to tackle the COVID-19 pandemic. Low perception of the virus susceptibility (26.3%) or severity (31.6%), and lack of perceived benefit of vaccination (32.2%) were amongst the common correlating factors of VH [46]. Trust is a well-known determinant of vaccine attitude that might have been threatened by ambiguous news often spread through social media [47]. Evidence suggests that children have shown lower susceptibility to COVID-19 infection [48], very few children developed severe COVID-19 (even if they were in a clinical risk group), and their role in the transmission of COVID-19 was unclear [44]. Likewise, with influenza vaccination, parents in our study have most likely perceived those benefits as outweighing the side effects and that their children would be safer if vaccinated [49].

## 6. Vaccine Acceptance among Previously Hesitant Parents

Twenty-three percent of hesitant parents have changed their minds within one month (up to 27 June 2021), and their young adolescents had either received at least one dose of the COVID-19 vaccine or booked an appointment; this can be considered as an indicator of raised public acceptance. Such findings could also be linked to the changes in Qatar policies imposed by the government as part of gradual 2021-year restriction lifting (restricted access to most public venues such as malls, cinemas, and restaurants, etc. solely for those vaccinated individuals including 12–15-year age group). This has probably brought about behavioural changes influencing VH over time. In fact, such regulations might help in providing a sense of route back to normality through public health and safety measures. Multisectoral efforts with combined pragmatic, holistic and sustainable messaging could contribute to building further public confidence. The emergence of highly contagious COVID-19 new variants such as Delta and Gamma [50], that are more likely to cause serious illness leading to hospital admission irrespective of patients’ age, could be a reason that led parents to change their minds in vaccinating their young adolescents [50].

## 7. Extension

Although the result of our study is promising in terms of relatively high coverage of BNT162b2 COVID-19 vaccination in Qatar, unfortunately, vaccine equity remains the only way out of this crisis. Failure to tackle structural and systematically inequalities in the acquisition and delivery of the COVID-19 vaccine, in addition to mutations in the COVID-19 virus, which can potentially render the first generation of vaccines ineffective in a year, coupled with ongoing travel will imply a fundamental threat toward ending this pandemic. The call for action by the WHO and the United Nations has urged all countries to invest in fair health systems that promote progress towards more effective containment, surveillance, and universal health coverage, ensuring that marginalized populations can access quality, affordable and comprehensive care. Furthermore, the observed low VH rate in our study is possibly influenced by the Qatar government providing the COVID-19 vaccines free of charge for both citizens and residents, diminishing potential barriers to vaccination. The socioeconomic status, vaccine availability, continuous supply, and storage requirements are all determinant factors that might jeopardize the coverage and success of vaccination programs. According to the WHO, lack of COVID-19 vaccine supply, injustice and inequitable distribution remains the biggest threat to the acute stage of this pandemic and driving a global recovery [51].

## 8. Strengths and Limitations

The main strengths of the study are the large cohort of enrolled participants rendering it representative of the local population: (i) the homogeneous distribution of the population (age, sex, and nationality), (ii) the absence of operator influence on the phone calls booking appointment in addition to the timing of the study, which started two days following the FDA vaccine approval (first-ever approval globally) for this age group. Another strength of this study is that COVID-19 vaccines have been obtainable free of charge for both citizens and residents as explained above. Indeed, the cost of the vaccine could constitute a barrier for co-variables such as the socioeconomic status of the individuals, vaccine availability, continuous supply as well as the storage requirements that could impact the VH rate in the present findings. This study has four main limitations. The first one is related to the use of retrospective data of phone calls for booking appointments. Indeed, there was no prior intention for publication of the outcomes related to this vaccine campaign, and consequently, the reason for parental denial of the vaccines amongst this age group is not enough well elaborated and detailed. Therefore, using the health belief model would provide a better understanding of the VH reasons. The second limitation is linked to parents’ demographic and education data as well as the adolescents’ vaccination history (routine childhood immunization) were not collected. Thus, this might have allowed a clear understanding of the rationales of the hesitancy of the BNT162b2 COVID-19 vaccine for this age group. Third, this study did not access the appointment outcome (actual uptake) and the no-show rate is unknown. Indeed, no-shows are a main concern for public health, which adversely impacts the workflow and efficiency of health care provided [52], and thus can negatively impact the desired outcome of the vaccine campaign. Finally, the recruitment of participants concerns one single health centre, leading to reduced generalizability of the study findings that may undermine its’ external validity [although it is one of the main COVID-19 (treatment and vaccination) dedicated facilities in the country central region, with a high influx of diverse population].

## 9. Conclusions

This study highlighted a low rate of VH amongst the whole population of Qatar for the vaccination of adolescents aged 12 to 15 years old. Nevertheless, there were unprecedented levels of VH amongst parents of young adolescents belonging to Gulf Countries (35.24%). Effective campaigns of health promotion awareness should target this group of nationalities to support efforts to vaccinate younger adolescents against SARS-CoV-2, potentially curtailing asymptomatic viral circulation and, hopefully, achieving herd immunity.

## Figures and Tables

**Table 1:** Characteristic of the participants (parents and young adolescents) and their association with the BNT162b2 COVID-19 vaccination booking status (*n* = 4023).

Characteristics	Total *n* = 4023 (100%)	Vaccine Booking Status		Multivariate Analysis (Binary Logistic Regression)		
		Acceptance *n* = 3303 (82.10%)	Hesitancy *n* = 720 (17.90%)	OR	95% CI	*p*-Value
**Adolescents’ Age Groups**						
12 years	1100 (27.34)	78.36	21.64	1.38	1.12–1.70	0.002
13 years	1100 (27.34)	84.00 ^a^	16.00	1.04	0.85–1.28	0.718
14 years	959 (23.84)	81.75	18.25	1.14	0.92–1.41	0.231
15 years	864 (21.48)	84.84 ^b^	15.16	REF		
Chi-2 test: *p*-value		0.0001 *				
**Sex**						
Boys	1983 (49.29)	82.25	17.75	0.98	0.85–1.13	0.775
Girls	2040 (50.71)	81.96	18.04	REF		
Chi-2 test: *p*-value		0.8104				
**Nationalities**						
Asiatic	2552 (63.44)	84.80	15.20	0.38	0.29–0.50	0.000
North-African	844 (20.98)	79.62 ^a^	20.38	0.48	0.36–0.65	0.000
African	199 (4.95)	82.91	17.09	0.41	0.27–0.62	0.000
E & A & O	113 (2.81)	86.73	13.27	0.34	0.21–0.56	0.000
GC (97% Qatari)	315 (7.83)	64.76 ^b–e^	35.24	REF		
Chi-2 test: *p*-value		0.0001 *				
**Chronic Disease**						
Yes	503 (12.50)	78.73	21.27	1.13	0.91–1.40	0.290
No	3520 (87.50)	82.59	17.41	REF		
Chi-2 test: *p*-value		0.0346 *				
**COVID-19^+ve^**						
Yes	253 (6.29)	75.89	24.11	1.37	1.02–1.84	0.036
No	3770 (93.71)	82.52	17.48	REF		
Chi-2 test: *p*-value		0.0077 *				

Data were number (%) for the total sample and % for the “vaccine booking status”; CI: confidence interval. COVID-19^+ve^: COVID-19 positive last 90 days. E & A & O: European/American/Oceanian. GC: Gulf Countries. OR: odds ratio. REF: reference. * *p* < 0.05 (Chi-2 test): comparison between 2 [sex (boys vs. girls), Chronic disease (no vs. yes), COVID-19^+ive^ (no vs. yes)], 4 (age groups) or 5 (nationalities) categories data. Age groups: ^a^ 12 vs. 13 years; ^b^ 12 vs. 15 years. Nationalities: ^a^ North-African vs. Asiatic; ^b^ GC vs. Asiatic, ^c^ GC vs. North-African, ^d^ GC vs. African, ^e^ GC vs. E & A & O. Binary logistic regression: dependent variable = vaccine booking status with “vaccine acceptance” as a reference category.

**Table 2:** Reasons for parental vaccine hesitancy (*n* = 720).

Characteristics	Wait and See *n* = 509 (70.69%)	Definitely Refusal *n* = 187 (25.97%)	Currently Sick/COVID-19^+ve^ *n* = 24 (3.34%)
**Adolescents’ Age Groups**			
12 years (*n* = 238)	176 (73.95)	59 (24.79)	3 (1.26)
13 years (*n* = 176)	117 (66.48)	50 (28.41)	9 (5.11)
14 years (*n* = 175)	129 (73.71)	40 (22.86)	6 (3.43)
15 years (*n* = 131)	87 (66.41)	38 (29.01)	6 (4.58)
Chi-2 test: *p*-value	0.21		
**Sex**			
Boys (*n* = 352)	248 (70.45)	89 (25.28)	15 (4.27)
Girls (*n* = 368)	261 (70.92)	98 (26.63)	9 (2.45)
Chi-2 test: *p*-value	0.38		
**Nationalities**			
Asiatic (*n* = 388)	287 (73.97)	91 (23.45)	10 (2.58)
North-African (*n* = 172)	115 (66.86)	52 (30.23)	5 (2.91)
African (*n* = 34)	24 (70.59)	8 (23.53)	2 (5.88)
E & A & O (*n* = 15)	10 (66.67)	5 (33.33)	0 (0.00)
GC (*n* = 111; 91% Qatari)	73 (65.77)	31 (27.93)	7 (6.30)
Chi-2 test: *p*-value	0.34		
**Chronic Disease**			
Yes (*n* = 107)	61 (57.01)	33 (30.84)	13 (12.15)
No (*n* = 613)	448 (73.08)	154 (25.12)	11 (1.80)
Chi-2 test: *p*-value	0.03 *^,a,b^		
**COVID-19^+ve^**			
Yes (*n* = 61)	39 (63.93)	6 (9.84)	16 (26.23)
No (*n* = 659)	470 (71.32)	181 (27.47)	8 (1.21)
Chi-2 test: *p*-value	0.02 *^,a,b^		

Data were number (%). COVID-19^+ve^: COVID-19 positive during the last 90 days. E & A & O: European/American/Oceanian. GC: Gulf Countries. * *p* < 0.05 (Chi-2 test): comparison between 2 [sex (boys vs. girls), Chronic disease (no vs. yes), COVID-19^+ive^ (no vs. yes)], 4 (age groups) or 5 (nationalities) categories data. Chronic disease: ^a^ Wait and see, ^b^ Currently sick or COVID-19^+ve^; COVID-19^+ve^: ^a^ Definitely refusal, ^b^ Currently sick or COVID-19^+ve^.

## Data Availability

Data are available from the authors (S.M., or I.D.) upon reasonable request.

## Supplementary Materials

The following are available online at https://www.mdpi.com/article/10.3390/vaccines9090981/s1. Table S1: Nationalities and vaccine acceptance rates per nationality of the parents of young adolescents aged 12–15 years.
