# Supplementary material for: BNT162b2 COVID-19 Vaccine Hesitancy among Parents of 4023 Young Adolescents (12–15 Years) in Qatar
Source: Vaccines (Basel). 2021 Sep 2;9(9):981. doi: 10.3390/vaccines9090981 (PMC8473301; doi:10.3390/vaccines9090981)
Supplement: Supplementary file 1 [file vaccines-09-00981-s001.zip › vaccines-1299317-supplementary.pdf]

**Table S1. Nationalities and vaccine acceptance rates per nationality of the parents of young adolescents aged 12-15 years.**								
**Nationality**	Number (%)	Acceptante rate (%)	**Nationality**	Number (%)r	Acceptante rate (%)	**Nationality**	Number (%)	Acceptante rate (%)
Indian	**1149 (28.57)**	88.51	Nigerian	6 (0.15)	-	Bosnian	1 (0.02)	-
Egyptian	**698 (17.35)**	80.37	Somali	5 (0.12)	-	Cameroonian	1 (0.02)	-
Filipino	**391 (9.72)**	87.72	Afghan	4 (0.10)	-	Chadian	1 (0.02)	-
Jordanian	**305 (7.58)**	72.46	Libyan	4 (0.10)	-	Cypriot	1 (0.02)	-
Qatari	**305 (7.58)**	65.25	Portuguese	4 (0.10)	-	Dominican	1 (0.02)	-
Pakistani	211 (5.25)	**92.42**	Ethiopian	3 (0.07)	-	Eritrean	1 (0.02)	-
Sudanese	178 (4.43)	82.58	Omani	3 (0.07)	-	Ghanaian	1 (0.02)	-
Syrian	105 (2.61)	75.24	Saudi	3 (0.07)	-	Italian	1 (0.02)	-
Lebanese	91 (2.26)	84.62	Spanish	3 (0.07)	-	Kuwaiti	1 (0.02)	-
Iranian	89 (2.21)	69.66	Albanian	2 (0.05)	-	Maldivian	1 (0.02)	-
Tunisian	84 (2.09)	76.19	Australian	2 (0.05)	-	Nicaraguan	1 (0.02)	-
Palestinian	79 (1.96)	72.15	Bahraini	2 (0.05)	-	Norwegian	1 (0.02)	-
Yemeni	49 (1.22)	71.43	Chilean	2 (0.05)	-	Salvadoran	1 (0.02)	-
Bangladeshi	31 (0.77)	93.55	Djiboutian	2 (0.05)	-	Serbian	1 (0.02)	-
American	29 (0.72)	89.66	Finnish	2 (0.05)	-	South African	1 (0.02)	-
British	28 (0.70)	85.71	Hungarian	2 (0.05)	-	South Korean	1 (0.02)	-
Algerian	22 (0.55)	**59.09**	Irish	2 (0.05)	-	Tanzanian	1 (0.02)	-
Indonesian	22 (0.55)	90.91	Kazakhs	2 (0.05)	-	**Total**	**4023 (100)**	
Malaysian	13 (0.32)	100	Netherlands	2 (0.05)	-			
Moroccan	11 (0.27)	81.82	New Zealander	2 (0.05)	-			
Iraqi	10 (0.25)	90.00	Polish	2 (0.05)	-			
Canadian	7 (0.17)	-	Singaporean	2 (0.05)	-			
Greek	7 (0.17)	-	Ukrainian	2 (0.05)	-			
Brazilian	6 (0.15)	-	Venezuelan	2 (0.05)	-			
French	6 (0.15)	-	Austrian	1 (0.02)	-			
German	6 (0.15)	-	Belizean	1 (0.02)	-			
Mauritanian	6 (0.15)	-	Bolivian	1 (0.02)	-			
Vaccine acceptance rates were calculated only for nationalities including ≥10 participants.								
